# Dental Department of the University of Maryland

**Published:** 1883-04

**Authors:** 


### Dental Department of the University of Maryland.

The first annual commencement of the Dental Department of the University of Maryland took place at the Academy of Music, Baltimore, on Thursday, March 15, 1883.

Reading of the mandamus and announcement of graduates by the Dean, Ferdinand J. S. Gorgas, M.D.,D.D.S.

The address was delivered by Hon. John V. L. Findlay.

The number of matriculates for the session was sixty-six.

The degree of D.D.S. was conferred on the following gentlemen, thirty-four in number, by Hon. S. Teackle Wallis, L.L.D., Provost of the University:

| NAME. | RESIDENCE. |
|---|---|
| J. Hardin Baldwin, | Kentucky. |
| F. Austin Banks, | Michigan. |
| Bartow B. Breedon, | South Carolina. |
| Paul Campbell, | New York. |
| Frank G. Conklin, | New York. |
| Joseph W. Curtis, | New Jersey. |
| Erastus S. Dashiell, | Maryland. |
| Newton W. Denton, | Virginia. |
| R. Delamer Dodson, | Pennsylvania. |
| John F. Garrett, | North Carolina. |
| Godfrey J. Grempler, | Maryland. |
| George W. Hotaling, | New York. |
| R. Arthur Hungerford, | Maryland. |
| Atwell T. Jarrett, | Virginia. |
| Geo. Wilfred Le Duke, | Massachusetts. |
| Charles T. Lindsey, | Virginia. |
| B. Frank Maphis, | Virginia. |
| Wattie B. McGirt, | South Carolina. |
| J. Edwin Miller, | Minnesota. |
| Eli. H. Neiman, | Pennsylvania. |
| A. Lee Pennel, | Maryland. |
| Walter W. Rowe, | Pennsylvania. |
| Hippolyte C. Salles, | Louisiana. |
| Carlos N. Sanchez, | Cuba. |
| C. Julian Smith, | South Carolina. |
| Walter O. Smith, | Virginia. |
| Myron W. Snyder, | New York. |
| Walter Stuart, | Kentucky. |
| Newton Addison Teague, | South Carolina. |
| Norman B. Tipton, | Louisiana. |
| J. Everett Toombs, | Massachusetts. |
| Geo. Andreas Vollck, | Maryland. |
| Fred. Allen Weaver, | Massachusetts. |
| Aug. F. L. Weitfeldt, | Germany. |

Number of matriculates, sixty-six—all of whom, without a single exception, were dental students, and not matriculated in the medical department. Number of graduates, 34—all of whom *attended the entire course.*

The University prize, a gold medal, was awarded to John F. Garrett, of North Carolina, for the highest number of votes at the final examinations; J. Edwin Miller, of Minnesota, receiving honorable mention.

The first S. S. White prize was awarded to F. Austin Banks, of Michigan; Frank G. Conklin, of Indiana, and J. Everett Toombs, of New York, receiving honorable mention. The Snowden and Cowman prize was awarded to G. A. Volck, of Maryland. The second S. S. White prize was awarded to Walter Stuart, of Kentucky. The DENTAL REGISTER prize was awarded to Frank G. Conklin, of South Bend, Ind. The Wilkerson prize was awarded to Myron W. Snyder, of New York. The Genesee prize was awarded to Eli. H. Neiman, of Pennsylvania. The *Southern Dental Journal* prize was awarded to Walter W. Rowe, of Pennsylvania.

